# Dual Sensor Control Scheme for Multi-Target Tracking

**DOI:** 10.3390/s18051653

**Published:** 2018-05-21

**Authors:** Wei Li, Chongzhao Han

**Affiliations:** MOE KLINNS Lab, Institute of Integrated Automation, School of Electronic and Information Engineering, Xi’an Jiaotong University, Xi’an 710049, China

**Keywords:** sensor control, POMDPs, multi-target tracking, FISST-based filter

## Abstract

Sensor control is a challenging issue in the field of multi-target tracking. It involves multi-target state estimation and the optimal control of the sensor. To maximize the overall utility of the surveillance system, we propose a dual sensor control scheme. This work is formulated in the framework of partially observed Markov decision processes (POMDPs) with Mahler’s finite set statistics (FISST). To evaluate the performance associated with each control action, a key element is to design an appropriate metric. From a task-driven perspective, we utilize a metric to minimize the posterior distance between the sensor and the target. This distance-related metric promotes the design of a dual sensor control scheme. Moreover, we introduce a metric to maximize the predicted average probability of detection, which will improve the efficiency by avoiding unnecessary update processes. Simulation results indicate that the performance of the proposed algorithm is significantly superior to the existing methods.

## 1. Introduction

Multi-target tracking (MTT) refers to the state estimation of an unknown number of moving targets. The measurements are subject to missed detections and clutter. Mahler’s finite set statistics (FISST) [1] has provided a unified framework to deal with the MTT problem. For the MTT problem with controllable sensors, it involves multi-target state estimation and optimal control of the sensor [2,3]. The sequential estimation and decision-making process constitute its main content. It has attracted intense interest in the modern surveillance system [4]. Surveys of recent advances in multi-target tracking and sensor control have been presented in [5,6].

The sensor control problem for multi-target tracking has been studied in the context of partially observed Markov decision processes (POMDPs) [7] with FISST. As a key element in the POMDPs, a predefined metric is used to evaluate the performance associated with each control action. Actually, the optimal control process is carried out before the real measurement is observed. Generally, the combination of an appropriate metric and an excellent filter will improve the overall performance.

To solve the above problem, scholars have developed a series of methods. Mahler has proposed the idea of using the Kullback–Leibler discrimination as a metric in [8]. Later, he has defined a metric as the posterior expected number of targets in [9]. Depending on the development of multi-target filters, Ristic, and Vo [10,11] have developed the α-divergence based sensor control algorithms via the probability hypothesis density filter [12,13]. Hoang and Vo [14] have used two objective functions via the Cardinality Balanced Multi-Target Multi-Bernoulli (CB-MeMBer) filter [15]. Gostar et al. have utilized the Cauchy–Schwarz divergence for the CB-MeMBer filter [16], etc. From an information theoretic viewpoint, the mechanism of such algorithm is to measure the information gain between the updated posterior densities and the predicted density [17,18]. In addition, several methods can be classified as the task-driven algorithms [9,14,18,19], and others can be classified as the information-driven algorithms [8,10,11,16]. Obviously, the previous works have paid enough attention to the choice of metrics (tasks or information divergences), while seldom focusing on the structure.

This paper was committed to improving the efficiency of sensor control algorithm and maximizing the overall utility of the surveillance system. The main contribution is to propose a dual sensor control scheme for the MTT problem. In addition, two novel metrics were developed for the sensor control process. By minimizing the posterior distance between sensor and targets (PDST), the sensor can be driven to the targets directly. By maximizing the predicted average probability of detection (PAPD), more reliable measurements are observed. From a task-driven perspective, both the PDST and the PAPD are based on the understanding of improving the performance of the surveillance system. In particular, the distance-related metric PDST contributes to the redesign of the structure. Moreover, the sensor control process using the PAPD metric is valid while avoiding unnecessary update steps. Typically, a dual sensor control scheme contains two controllers, in which the metric pair is composed to distinguish different algorithms. Furthermore, the existing evaluation functions can be applied to the proposed dual sensor control scheme directly.

The remaining part of this article is structured as follows. A general formulation of the sensor control process is given in Section 2. For completeness, we present a brief review of the δ-Generalized labeled multi-Bernoulli (GLMB) filter in Section 3. Section 4 contains the main work of the proposed strategies, and Section 5 describes the dual sensor control algorithms for MTT with one controllable sensor via δ-GLMB filter. Simulation results and analysis are given in Section 6. Conclusions are drawn in Section 7.

## 2. The Formulation of Sensor Control

For a general nonlinear multi-target tracking system with the sensor control problem, we have

(1) $$\begin{array}{ccc} X_{k} & = & f_{k}\left(X_{k-1}\right)+w_{k}, \end{array}$$

(2) $$\begin{array}{ccc} Z_{k} & = & h_{k}\left(X_{k},S_{k}\right)+v_{k}, \end{array}$$

(3) $$\begin{array}{ccc} S_{k} & = & q_{k}\left(S_{k-1},U_{k}\right)+r_{k}, \end{array}$$

where $f_{k}$ denotes an evolution model, $h_{k}$ denotes an observation model, and $q_{k}$ denotes a control model. *X* represents the state of targets, *Z* represents the measurement, *S* represents the state of sensors, and *U* represents the selected control actions. The noise is used to describe the uncertainty. This problem can be roughly divided into three parts: *Filter*, *Observer*, and *Controller*. These three parts interact with each other.

**Figure 1:**
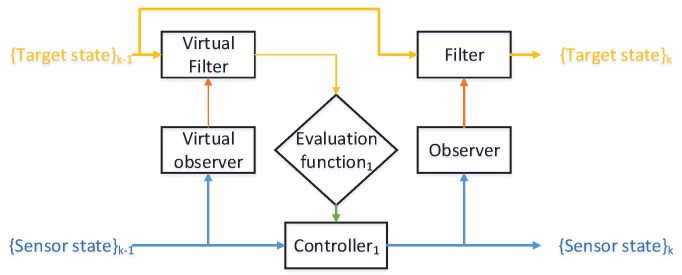
A general diagram of the sensor control process. (yellow lines represent the data flow of targets’ states; blue lines represent sensors’ states; red lines are the observations; green lines represent for the decision-making process).

Figure 1 illustrates a general diagram of the sensor control process. Generally, this process is carried out in the framework of the POMDPs [2]. The key elements of a POMDP include:

1. a portrayal of the multi-target posterior probability density function (pdf);
2. the admissible control actions of the sensors;
3. a predefined metric works to evaluate various control actions.

The following parts of this section illustrate the three aspects specifically.

### 2.1. Bayesian Multi-Target Filtering

Mahler’s FISST has provided a batch of solutions to the MTT problem in the random finite set (RFS) framework, and the methodologies throughout this paper are derived in this background. Following the conventional notation, we use small letters to denote the single-target states, e.g., x, z while the capital letters for the multi-target states, e.g., *X*, *Z*. In addition, blackboard bold letters denote spaces, e.g., X, Z. $F\left(X\right)$ represents the collection of all finite sets of the space X. At time *k*, we have the following RFS descriptions of a multi-target state and a multi-target measurement:

(4) $$\begin{array}{ccc} X_{k} & = & \left\{x_{k,1},\ldots ,x_{k,n_{k}}\right\}\in F\left(X\right), \end{array}$$

(5) $$\begin{array}{ccc} Z_{k} & = & \left\{z_{k,1},\ldots ,z_{k,m_{k}}\right\}\in F\left(Z\right), \end{array}$$

where $X_{k}$ in Equation (4) encapsulates the target motions, births, and deaths. In addition, $Z_{k}$ in Equation (5) encapsulates the imperfect detection and false alarms. Let $\pi_{k|k-1}\left(X_{k}|Z_{1:k-1}\right)$ denote the predicted multi-target posterior density, and $\pi_{k}\left(X_{k}|Z_{1:k}\right)$ denote the updated multi-target posterior density at *k*. Then, the predicted and updated multi-target posterior densities are calculated as follows:

(6) $$\begin{array}{ccc} \pi_{k|k-1}\left(X_{k}|Z_{1:k-1}\right) & = & \int f_{k}\left(X_{k}|X\right)\pi_{k-1}\left(X|Z_{1:k-1}\right)\delta X, \end{array}$$

(7) $$\begin{array}{ccc} \pi_{k}\left(X_{k}|Z_{1:k}\right) & = & \frac{g_{k}\left(Z_{k}|X_{k}\right)\pi_{k|k-1}\left(X_{k}|Z_{1:k-1}\right)}{\int g_{k}\left(Z_{k}|X\right)\pi_{k|k-1}\left(X|Z_{k}\right)\delta X}, \end{array}$$

where $f_{k}\left(X_{k}|X\right)$ is the multi-target transition density and $g_{k}\left(Z_{k}|X_{k}\right)$ is the multi-target likelihood function. Generally, the multi-target posteriors can be computed sequentially via the prediction and the update steps.

### 2.2. Ideal Control Process

For the sensor control problem with a fixed number of controllable sensors, the state of the sensors can be represented by

(8) $$S_{k}=\left\{s_{k,1},\ldots ,s_{k,i},\ldots ,s_{k,s}\right\}.$$

A general task of the sensor control problem is to determine the optimal control action for each sensor. Let $U_{k}\in U_{k}$ denote the desired optimal control action and $U_{k}$ be the admissible control actions. Then,

(9) $$U_{k}=\left\{u_{k,1},\ldots ,u_{k,i},\ldots ,u_{k,s}\right\},$$

where $u_{k,i}$ denotes the optimal control action for the *i*th sensor.

Most of the existing methods use an ideal control process (ICP) for simplification, in which each sensor can be driven to several positions without considering the specific dynamic process. Therefore, the admissible control actions are quantified. Given the previous positions of the sensors $S_{k-1}$, their one-step ahead positions are adopted as

(10) $$S_{k}=S_{k}\left(S_{k-1},U_{k}\right)\in S_{k}\left(S_{k-1},U_{k}\right),$$

where $S_{k}\left(S_{k-1},U_{k}\right)$ denote the admissible positions.

Actually, the term admissible control actions and the admissible positions of the sensors are equivalent in the context of ICP. For instance, we can define a set of admissible control actions as [10]

(11) $$U_{k,i}=\left\{\left(x_{k-1,i}+j\frac{v_{i}T}{n_{R}}\cos\left(l\frac{2\pi }{n_{\theta }}\right),y_{k-1,i}+j\frac{v_{i}T}{n_{R}}\sin\left(l\frac{2\pi }{n_{\theta }}\right)\right)\right\},$$

where ${\left\{\left(x_{k-1,i},y_{k-1,i}\right)\right\}}_{i=1}^{s}$ represents for the previous positions of the sensors, $v_{i}$ can be viewed as the maximum speed of the *i*th sensor, $j=0,1,\cdots ,n_{R}$ denotes the variety of the speeds, and $l=1,\cdots ,n_{\theta }$ denotes the variety of directions. $n_{R}$ and $n_{\theta }$ are set to be constant. T is the step length of the control process, and we set T=1 in this paper to represent the single step control process.

**Figure 2:**
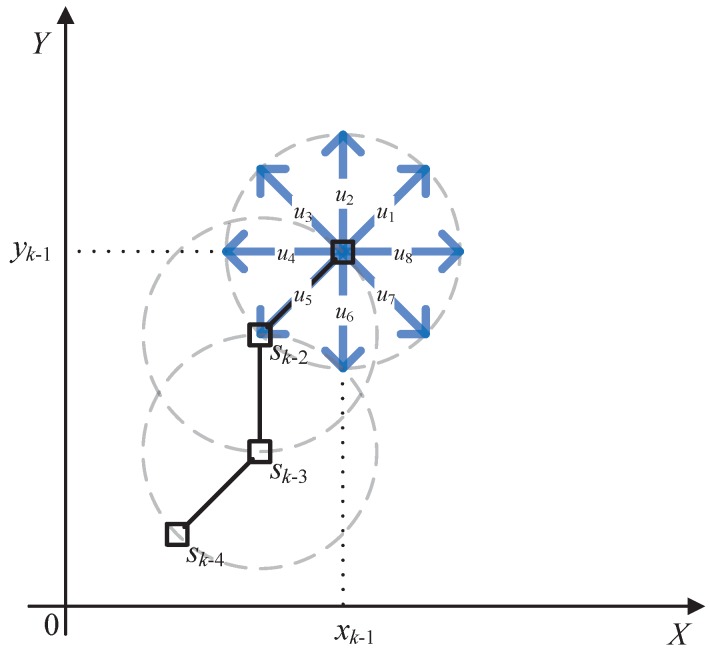
The trajectory of a sensor using ICP. The black □ denotes the position of the sensor; the blue → denotes admissible control actions; and the black solid line denotes the trajectory.

Figure 2 gives an example of a single sensor situation with parameters s=1, $j=n_{R}=1$, and $n_{\theta }=8$. The speed *v* is equivalent to the length of the arrow, and the endpoints represent the admissible positions of the sensor. Once the optimal control action $u_{k}$ is determined, we can drive the sensor to one of the eight positions.

### 2.3. Evaluation Function

Let $E^{1}\left(\cdot \right)$ denote a reward function. An optimal control strategy is formulated as [10]

(12) $$U_{k}^{1}=\arg\max_{U\in U_{k}^{1}}E\left[E^{1}\left(U,\pi_{k-1}\left(X_{k-1}|Z_{1:k-1}\right),Z_{k}^{1}\left(U,S_{k-1}\right)\right)\right],$$

where $U_{k}^{1}$ is the admissible control actions, $\pi_{k-1}\left(X_{k-1}|Z_{1:k-1}\right)$ is the previous updated multi-target posterior density calculated by Equations (6) and (7), and $Z_{k}^{1}\left(U,S_{k-1}\right)$ is a virtual observation associated with a specific control action. The optimal control action is selected by maximizing this expectation E[·] in ${\mathrm{Controller}}_{1}$.

From a POMDP perspective, the reward function $E^{1}\left(U,\pi ,Z\right)$ is a real-valued function associated with the control *U*, the previous posterior pdf π, and the current measurement *Z*. In fact, the current measurement $Z_{k}$ used by the *Filter* can only be observed after applying the sensor control process. Therefore, the virtual measurement $Z_{k}^{1}\left(U,S_{k-1}\right)$ is involved. Once the optimal control action is determined, we can drive the sensors to the new positions $S_{k}^{1}$.

The previous studies have demonstrated that the predefined metric (reward function or cost function) plays a very important role in the POMDPs. It is necessary to develop an efficient task-driven strategy for the specific problem.

### 2.4. Predicted Ideal Measurement

Recall the virtual measurement $Z_{k}^{1}\left(U,S_{k-1}\right)$ in Equation (12). The predicted ideal measurement (PIM) is introduced to substitute the missing measurement for its virtual update step. For example, a PIM can be generated by taking the predicted state into the observation model $h_{k}$ in Equation (2). Generally, different control actions will generate different PIMs. The validity of using the PIMs implies that the observation model is accurate, which is a common assumption held by most existing methods.

## 3. Delta-Generalized Labeled Multi-Bernoulli Filter

For the multi-target state estimation, this part provides a brief review of δ-Generalized labeled multi-Bernoulli filter filter (GLMB) [20,21]. Following the conventional notations in the references, a δ-GLMB is completely characterized by the set ${\left\{\left(\omega^{\left(I,\xi \right)},p^{\left(\xi \right)}\left(\cdot ,\cdot \right)\right)\right\}}_{I\subseteq L,\xi \in \Xi }$. The δ-GLMB filter propagates a δ-GLMB density through prediction and update steps recursively. For simplification, we omit the time index and use the symbol “+” to denote the predicted quantities.

### 3.1. Prediction

The predicted density are combined with two parts, the existing density and the newborn density. Assume that the birth process is formulated as a GLMB RFS in X×B, where B is the label space of newborn targets. For the existing density, the label space is L. The two space should be distinct, i.e., L∩B=∅.

The labeled multi-Bernoulli birth model is

(13) $$\begin{array}{ccc} \omega_{B}\left(L\right) & = & \prod_{l\in B}\left(1-\gamma_{B}^{\left(l\right)}\right)\prod_{l\in L}\frac{1_{B}\left(l\right)\gamma_{B}^{\left(l\right)}}{\left(1-\gamma_{B}^{\left(l\right)}\right)}, \end{array}$$

(14) $$\begin{array}{ccc} p_{B}\left(x,l\right) & = & p_{B}^{\left(l\right)}\left(x\right). \end{array}$$

For the existing part, we have the δ-GLMB parameter set ${\left\{\left(\omega^{\left(I,\xi \right)},p^{\left(\xi \right)}\left(\cdot ,\cdot \right)\right)\right\}}_{I\subseteq L,\xi \in \Xi }$. By using the notations $p_{S}\left(\cdot ,l\right)$ and $f\left(x|\cdot ,l\right)$ to denote the single-target survival probability and Markov transition density, the parameters of the survival δ-GLMB are

(15) $$\begin{array}{ccc} \omega_{S}^{\left(\xi \right)}\left(L\right) & = & {\left[\eta_{S}^{\left(\xi \right)}\right]}^{L}\sum_{L\subseteq I\subseteq L}{\left[1-\eta_{S}^{\left(\xi \right)}\right]}^{I-L}\omega^{\left(I,\xi \right)}, \end{array}$$

(16) $$\begin{array}{ccc} p_{S}^{\left(\xi \right)}\left(x,l\right) & = & \frac{\langle p_{S}\left(\cdot ,l\right)f\left(x|\cdot ,l\right),p^{\left(\xi \right)}\left(\cdot ,l\right)\rangle }{\eta_{S}^{\left(\xi \right)}\left(l\right)}, \end{array}$$

(17) $$\begin{array}{ccc} \eta_{S}^{\left(\xi \right)}\left(l\right) & = & \langle p_{S}\left(\cdot ,l\right),p^{\left(\xi \right)}\left(\cdot ,l\right)\rangle , \end{array}$$

where the notation $\langle f,g\rangle \overset{\Delta }{\;=}\int f\left(x\right)g\left(x\right)dx$ denotes the inner product.

Given the current δ-GLMB multi-target posterior and the birth density, the predicted multi-target posterior to the next time is a δ-GLMB with parameter set ${\left\{\left(\omega_{+}^{\left(I_{+},\xi \right)},p_{+}^{\left(\xi \right)}\left(\cdot ,\cdot \right)\right)\right\}}_{I_{+}\subseteq L_{+},\xi \in \Xi }$, where $L_{+}=L\cup B$ denotes the new label space. The predicted δ-GLMB parameters are calculated by

(18) $$\begin{array}{ccc} \omega_{+}^{\left(I_{+},\xi \right)} & = & \omega_{S}^{\left(\xi \right)}\left(I_{+}\cap L\right)\omega_{B}\left(I_{+}\cup L\right), \end{array}$$

(19) $$\begin{array}{ccc} p_{+}^{\left(\xi \right)}\left(x,l\right) & = & 1_{L}\left(l\right)p_{S}^{\left(\xi \right)}\left(x,l\right)+1_{B}\left(l\right)p_{B}\left(x,l\right). \end{array}$$

### 3.2. Update

In the update step, the δ-GLMB filter takes missed detection and clutter into account. Thus, the probability of detection is denoted as $p_{D}\left(x,l\right)$ if detected. Recalling the notation of Equation (5), we use *Z* to denote the measurements set. For a measurement z, the clutter is assumed to be a Poisson RFS with intensity $\kappa \left(z\right)$. In addition, the likelihood of the measurement is denoted as $g\left(z|x,l\right)$. The parameters of the updated posterior ${\left\{{\left\{\left(\omega^{\left(I_{+},\xi ,\theta \right)}\left(Z\right),p^{\left(\xi ,\theta \right)}\left(\cdot ,\cdot |Z\right)\right)\right\}}_{\theta \in \Theta \left(I_{+}\right)}\right\}}_{I_{+}\subseteq L_{+},\xi \in \Xi }$ are given by

(20) $$\omega^{\left(I_{+},\xi ,\theta \right)}\left(Z\right)\;\;\propto \;\;\omega_{+}^{\left(I_{+},\xi \right)}{\left[\eta_{Z}^{\left(\xi ,\theta \right)}\left(l\right)\right]}^{I+},$$

(21) pξ,θZ=p+ξx,lψZx,l;θηZξ,θl,

(22) $$\begin{array}{ccc} \eta_{Z}^{\left(\xi ,\theta \right)}\left(l\right) & = & \langle p_{+}^{\left(\xi \right)}\left(\cdot ,l\right)\psi_{Z}\left(\cdot ,l;\theta \right)\rangle . \end{array}$$

The intermediate terms are

(23) ψZx,l;θ=1−pDx,l,ifθl=0,pDx,lgzθl|x,lκzθl,otherwise,

where $\theta \in \Theta (I_{+})$ is the association between the label set and the measurements.

### 3.3. State Estimation

From an implementation viewpoint, the multi-target state estimation is the purpose of the sensor chasing algorithm. In this part, we use a Marginal multi-Bernoulli estimator to extract the states, and the core of this idea is to extract estimates via best cardinality. The distribution of cardinality is

(24) $$\rho \left(n\right)=\sum_{\left(I,\xi \right)\in F_{n}\left(L\right)\times \Xi }\omega^{\left(I,\xi \right)},$$

where $n=1,...,N_{\max}$, and $F_{n}\left(L\right)$ denotes the subsets of space L with *n* targets, where $N_{\max}$ is the predefined maximum number of targets. The simplified estimation process is

(25) $$\begin{array}{ccc} \hat{N} & = & \arg\max\rho \left(n\right), \end{array}$$

(26) $$\begin{array}{ccc} \hat{X} & = & \left\{\left(x,l\right):l\in I^{\left(\hat{h},\hat{j}\right)},x=\int yp^{\left(\hat{h},\hat{j}\right)}\left(y,l\right)dy\right\}, \end{array}$$

where $\left(\hat{h},\hat{j}\right):=\arg\max_{\left(h,j\right)}\omega^{\left(h,j\right)}\delta_{\hat{N}}\left(\left|I^{\left(h,j\right)}\right|\right),$ which means we try to find the labels and states from the highest weighted element that has the cardinality $\hat{N}$.

## 4. The Proposed Strategies

### 4.1. A Novel Structure of Dual Sensor Control Scheme

**Figure 3:**
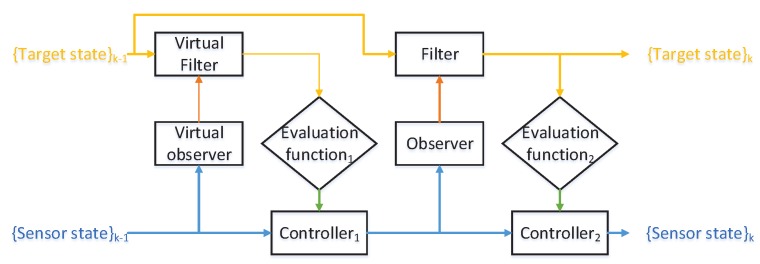
The proposed sensor control structure.

For most existing works, once the control action is determined, no further correction step is involved. To maximize the overall utility of the system, an additional decision-making process is introduced. In this part, we propose a dual sensor control scheme. Figure 3 shows a diagram of the proposed structure.

Let $E^{2}\left(\cdot \right)$ denote the evaluation function related to the ${\mathrm{Controller}}_{2}$, and the additional control process is

(27) $$U_{k}^{2}=\arg\max_{U^{2}\in U_{k}^{2}}E\left[E^{2}\left(U^{2},\pi_{k}\left(X_{k}|Z_{1:k}\right),S_{k}\left(U^{2},S_{k}^{1}\right)\right)\right],$$

where $U_{k}^{2}$ is the admissible control actions, and $\pi_{k}\left(X_{k}|Z_{1:k}\right)$ is the updated multi-target posterior density after applying the current measurement $Z_{k}$. $S_{k}\left(U^{2},S_{k}^{1}\right)$ is a one-step ahead position of the sensors associated with a control action $U^{2}$, and $S_{k}^{1}$ is the positions of the sensors after ${\mathrm{Controller}}_{1}$. The optimal control action is selected by maximizing this expectation E[·] in the ${\mathrm{Controller}}_{2}$.

Compared to the sensor control process in Equation (12), the evaluation function $E^{2}\left(U,\pi ,S\right)$ is a real-valued function associated with the control *U*, the current posterior pdf π, and the future positions of the sensors *S*. In fact, the additional sensor control process is carried out by utilizing the real measurement information. Similar to the ${\mathrm{Controller}}_{1}$, once the optimal control action is determined, we can drive the sensors to the new positions $S_{k}$.

### 4.2. Minimize the Posterior Distance between Sensor and Targets

For ${\mathrm{Controller}}_{2}$, an intuitive idea is to choose the control action that minimizes the distance between sensors and targets after getting the real observation. Therefore, we define a distance-related metric, namely the posterior distance between sensor and targets (PDST). Then, Equation (27) turns out to be

(28) $$\begin{array}{c} U_{k}^{2}=\arg\min_{U^{2}\in U_{k}^{2}}E\left[D\left({\hat{X}}_{k}\left(\pi_{k}\left(X_{k}|Z_{1:k}\right)\right),S_{k}\left(U^{2},S_{k}^{1}\right)\right)\right], \end{array}$$

where ${\hat{X}}_{k}\left(\cdot \right)$ is the estimated state of the targets extracted from the updated multi-target posterior density $\pi_{k}\left(X_{k}|Z_{1:k}\right)$. In addition, $S_{k}\left(U^{2},S_{k}^{1}\right)$ is a one-step ahead position of the sensors associated with a control action $U^{2}$.

Generally, the PDST is calculated between two sets with different cardinalities. As an example, we recommend utilizing the optimal sub-pattern assignment (OSPA) [22] metric. The OSPA distance between two sets $X=\{x_{1},\cdots ,x_{m}\}$ and $\hat{X}=\left\{{\hat{x}}_{1},\cdots ,{\hat{x}}_{n}\right\}$ is defined by

(29) $${\bar{d}}_{p}^{\left(c\right)}={\left(\frac{1}{n}\left(\min_{\pi \in \Pi_{n}}\sum_{i=1}^{m}d^{\left(c\right)}{\left(x_{i},{\hat{x}}_{\pi (i)}\right)}^{p}+c^{p}\left(n-m\right)\right)\right)}^{\frac{1}{p}},$$

where $d^{\left(c\right)}\left(x,\hat{x}\right):=\min\left(c,∥x-\hat{x}∥\right)$, $\Pi_{k}$ is the set of permutations on $\left\{1,2,\ldots ,k\right\}$, and the positive integer p≥1 and c>0. Given the estimation ${\hat{X}}_{k}\left(\cdot \right)=\left\{{\hat{x}}_{k,1},\ldots ,{\hat{x}}_{k,{\hat{N}}_{k}}\right\}$ and an implementation of the $S_{k}\left(U^{2},S_{k}^{1}\right)=\left\{s_{k,1},\ldots ,s_{k,i},\ldots ,s_{k,s}\right\}$, the PDST $D\left({\hat{X}}_{k}\left(\cdot \right),S_{k}\left(U^{2},S_{k}^{1}\right)\right)$ is adopted as ${\bar{d}}_{p}^{\left(c\right)}$ by substituting $X={\hat{X}}_{k}\left(\cdot \right)$ and $\hat{X}=S_{k}\left(U^{2},S_{k}^{1}\right)$.

An equivalent sensor control strategy can also be designed for ${\mathrm{Controller}}_{1}$ by using the PDST metric. Based on the output of the *Virtual Filter*, we can get the virtual updated multi-target posterior density $\pi_{k}^{1}\left(X_{k}^{1}|Z_{k}^{1}\left(U,S_{k-1}\right)\right)$, Then, the corresponding control equation is

(30) $$\begin{array}{c} U_{k}^{1}=\arg\min_{U^{1}\in U_{k}^{1}}E\left[D\left({\hat{X}}_{k}^{1}\left(\pi_{k}^{1}\left(X_{k}^{1}|Z_{k}^{1}\left(U^{1},S_{k-1}\right)\right)\right),S_{k}^{1}\left(U^{1},S_{k-1}\right)\right)\right], \end{array}$$

where ${\hat{X}}_{k}^{1}\left(\cdot \right)$ is the estimated state of the targets extracted from the virtual updated multi-target posterior density $\pi_{k}^{1}\left(X_{k}^{1}|Z_{k}^{1}\left(U,S_{k-1}\right)\right)$, which is produced by the *Virtual Filter*. Compared to the ${\hat{X}}_{k}\left(\cdot \right)$ in Equation (28), the main difference is the value of ${\hat{X}}_{k}^{1}\left(\cdot \right)$ is associated with the control action $U^{1}$. $S_{k}^{1}\left(U^{1},S_{k-1}\right)$ is a one-step ahead position of the sensors associated with a control action $U^{1}$.

Actually, the metrics for the two controllers do not need to be the same. Next, we are committed to developing a more efficient metric for ${\mathrm{Controller}}_{1}$.

### 4.3. Maximize the Predicted Average Probability of Detection

Since the computational mechanism of the existing evaluation functions depends on the virtual updated multi-target posterior densities, the *Virtual Filter* has to be employed several times. Obviously, it is very time-consuming in the ${\mathrm{Controller}}_{1}$.

In order to improve the efficiency, we define a novel evaluation function, namely the predicted average probability of detection (PAPD) metric. Equation (12) is simplified as

(31) $$\begin{array}{c} U_{k}^{1}=\arg\max_{U^{1}\in U_{k}^{1}}E\left[P_{D}\left({\hat{X}}_{k|k-1}\left(\pi_{k|k-1}\left(X_{k}|Z_{1:k-1}\right)\right),S_{k}^{1}\left(U^{1},S_{k-1}\right)\right)\right], \end{array}$$

where ${\hat{X}}_{k|k-1}\left(\cdot \right)$ is the estimated state of the targets extracted from the predicted multi-target posterior density $\pi_{k|k-1}\left(X_{k}|Z_{1:k-1}\right)$, which is calculated by Equation (6). $S_{k}^{1}\left(U^{1},S_{k-1}\right)$ is a one-step ahead position of the sensors associated with a control action $U^{1}$. Since the calculation of the PAPD metric $P_{D}\left(\cdot \right)$ is carried out based on the prediction, the update step is avoided. In addition, there is no need to involve the PIMs. Consequently, the *Virtual Observer* and the *Virtual Filter* in Figure 3 are reduced to a *Predictor*.

For a comprehensive understanding, the following section describes the main steps of the proposed dual sensor control algorithms.

## 5. Dual Sensor Control Algorithms

Following the structure in Figure 3, we present the details of the dual sensor control algorithms for the MTT problem with one controllable sensor via δ-GLMB filter. We use a metric pair to distinguish different algorithms. Algorithm 1 shows the pseudo-code of the dual sensor control algorithms.

**Algorithm 1** Dual sensor control algorithms**Input**: sensor position $s_{k-1}$,the posterior pdf $\pi_{k-1}$, and admissible control set $U_{k}$1. *Prediction*: compute the predicted pdf $\pi_{k|k-1}$ by Section 3.1${\mathrm{Controller}}_{1}$:**If** Metric == PAPD2.       *State estimation*: extracted the predicted estimated targets’ state ${\hat{X}}_{k|k-1}$ by Section 3.33.       For $u^{1}\in U_{k}^{1}$4.             Compute the admissible sensor position $s_{k}^{1}\left(u^{1},\;s_{k-1}\right)$ by Section 2.25.             *Evaluation*: maximize the PAPD by Equation (32)6.       Endfor: obtain the optimal control $u_{k}^{1}$, and drive the sensor to the new position $s_{k}^{1}\left(u_{k}^{1},s_{k-1}\right)$**Elseif** Metric == PDST7.       For $u^{1}\in U_{k}^{1}$8.             *PIMs*: generate the virtual observation $Z_{k}^{1}\left(u^{1}\right)$ by Section 2.49.             *Update*: compute the virtual updated posterior pdf $\pi_{k}^{1}\left(u^{1}\right)$ by Section 3.210.            *State estimation*: extract the virtual estimated targets’ state ${\hat{X}}_{k}^{1}\left(u^{1}\right)$ by Section 3.311.            Calculate the center of the virtual estimation $\frac{1}{{\hat{N}}_{k,u^{1}}^{1}}\sum_{i=1}^{{\hat{N}}_{k,u^{1}}^{1}}\left[{\hat{x}}_{k,i}^{1}\left(u^{1}\right)\right]$12.            Calculate the admissible sensor position $s_{k}^{1}\left(u^{1},s_{k-1}\right)$ by Section 2.213.            *Evaluation*: minimize the PDST by Equation (34)14.      Endfor: obtain the optimal control $u_{k}^{1}$, and drive the sensor to the new position $s_{k}^{1}\left(u_{k}^{1},\;s_{k-1}\right)$
**End**
**Observer**: get the real observation $Z_{k}$15. *Update*: compute the posterior pdf $\pi_{k}$ by Section 3.216. *State estimation*: extract the estimated targets’ state ${\hat{X}}_{k}$ by Section 3.3${\mathrm{Controller}}_{2}$:17. Calculate the center of the estimation $\frac{1}{{\hat{N}}_{k}}\sum_{j=1}^{{\hat{N}}_{k}}\left[{\hat{x}}_{k,j}\right]$18. For $u^{2}\in U_{k}^{2}$19.      Calculate the admissible sensor position $s_{k}^{2}\left(u^{2},\;s_{k}^{1}\left(u_{k}^{1},\;s_{k-1}\right)\right)$ by Section 2.220.      *Evaluation*: minimize the PDST by Equation (35)21. Endfor: obtain the optimal control $u_{k}^{2}$, and drive sensor to the new position $s_{k}^{2}\left(u_{k}^{2},\;s_{k}^{1}\left(u_{k}^{1},\;s_{k-1}\right)\right)$**Output**: control pair $\left\{u_{k}^{1},u_{k}^{2}\right\}$, sensor position $s_{k}$, the posterior pdf $\pi_{k}$, and the estimation ${\hat{X}}_{k}$

### 5.1. Dual Sensor Control Algorithm with PAPD and PDST

In this part, we set the PDST as the metric in the ${\mathrm{Controller}}_{1}$. At time *k*, input the previous position of the sensor $s_{k-1}$, the representation of the previous posterior density $\pi_{k-1}$, and the set of admissible control actions $U_{k}$. The output is the optimal control pair $\left\{u_{k}^{1},\;u_{k}^{2}\right\}$, the position of the sensor $s_{k}$, and the representation of the resulting posterior density $\pi_{k}$.

Let ${\hat{X}}_{k|k-1}=\left\{{\hat{x}}_{k|k-1,1},\ldots ,{\hat{x}}_{k|k-1,{\hat{N}}_{k|k-1}}\right\}$ denote the predicted multi-target state and ${\hat{X}}_{k}=\left\{{\hat{x}}_{k,1},\ldots ,{\hat{x}}_{k,{\hat{N}}_{k}}\right\}$ denote the posterior multi-target state. For a given $p_{D}\left(\cdot \right)$ function, the metric pair for Algorithm 1 is calculated as

(32) $$\begin{array}{ccc} u_{k}^{1} & = & \arg\max_{u^{1}\in U_{k}^{1}}\frac{1}{{\hat{N}}_{k|k-1}}\sum_{i=1}^{{\hat{N}}_{k|k-1}}\left[p_{D}\left({\hat{x}}_{k|k-1,i},s_{k}^{1}\left(u^{1},s_{k-1}\right)\right)\right], \end{array}$$

(33) $$\begin{array}{ccc} u_{k}^{2} & = & \arg\min_{u^{2}\in U_{k}^{2}}D\left(\frac{1}{{\hat{N}}_{k}}\sum_{j=1}^{{\hat{N}}_{k}}\left[{\hat{x}}_{k,j}\right],s_{k}^{2}\left(u^{2},s_{k}^{1}\left(u_{k}^{1},s_{k-1}\right)\right)\right), \end{array}$$

where D(·) is the Euclidean distance between the sensor and the center of the posterior multi-target state $\frac{1}{{\hat{N}}_{k}}\sum_{j=1}^{{\hat{N}}_{k}}\left[{\hat{x}}_{k,j}\right]$ in ${\mathrm{Controller}}_{2}$. Each time, the optimal control is determined, and the sensor will be driven to a new location. At time *k*, the final position of sensor is $s_{k}=s_{k}^{2}\left(u_{k}^{2},s_{k}^{1}\left(u_{k}^{1},s_{k-1}\right)\right)$.

### 5.2. Dual Sensor Control Algorithm with PDST and PDST

In this part, we set the PDST as the metric in the ${\mathrm{Controller}}_{1}$. Compared to the above formulas, the main difference is in the ${\mathrm{Controller}}_{1}$. Recalling the term in Section 2.4, PIMs are involved in the *Virtual Filter*. Due to the mechanism of a PIM generation, the result of the *Virtual Filter* changes with different control actions. Hence, the metric pair for Algorithm 1 is calculated as

(34) $$\begin{array}{ccc} u_{k}^{1} & = & \arg\min_{u^{1}\in U_{k}^{1}}D\left(\frac{1}{{\hat{N}}_{k,u^{1}}^{1}}\sum_{i=1}^{{\hat{N}}_{k,u^{1}}^{1}}\left[{\hat{x}}_{k,i}^{1}\left(u^{1}\right)\right],s_{k}^{1}\left(u^{1},s_{k-1}\right)\right), \end{array}$$

(35) $$\begin{array}{ccc} u_{k}^{2} & = & \arg\min_{u^{2}\in U_{k}^{2}}D\left(\frac{1}{{\hat{N}}_{k}}\sum_{j=1}^{{\hat{N}}_{k}}\left[{\hat{x}}_{k,j}\right],s_{k}^{2}\left(u^{2},s_{k}^{1}\left(u_{k}^{1},s_{k-1}\right)\right)\right), \end{array}$$

where ${\hat{N}}_{k,u^{1}}^{1}=\left|{\hat{X}}_{k}^{1}\left(u^{1}\right)\right|$ denotes the number of virtual estimated targets associated with control $u^{1}$.

Note that the structure of the proposed algorithm does not depend on a specific filter. When it comes to the dual sensor control scheme, the evaluation function for ${\mathrm{Controller}}_{2}$ is specified to be a PDST metric. In addition, single sensor control counterparts of the above algorithms can be easily implemented by omitting the ${\mathrm{Controller}}_{2}$.

## 6. Simulations

### 6.1. Setup of the Simulations

**Figure 4:**
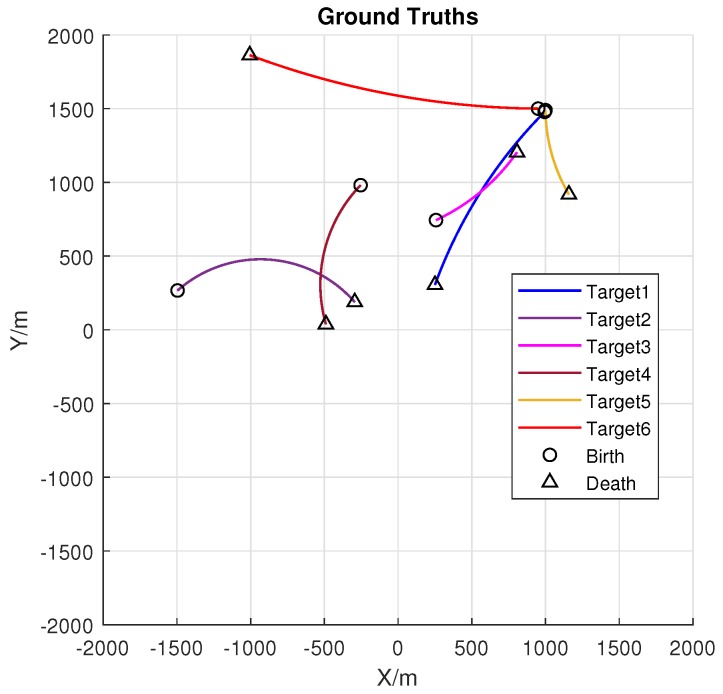
The ground truths of the simulations. (in total, six targets are involved with different colors to distinguish).

A nonlinear multi-target scenario is studied in this section. The number of targets varies over time, and the observations are affected by imperfect detection and clutter. Figure 4 is the ground truths of targets with a total of six targets in the surveillance area of 4000m×4000m.

The survival time of each target during the simulations: Target1 from 1 to 100; Target2 from 10 to 100; Target3 from 20 to 100; Target4 from 40 to 100; Target5 40 to 100; and Target6 from 60 to 100. The single-target state $x_{k}={\left[{\left({\tilde{x}}_{k}\right)}^{\prime },w_{k}\right]}^{\prime }$ is comprised of the location and velocity ${\tilde{x}}_{k}={\left[x_{k},{\dot{x}}_{k},y_{k},{\dot{y}}_{k}\right]}^{\prime }$ and the turning rate $w_{k}$. In addition the single-target transition density is defined as

$$f_{k|k-1}\left(x|x_{k}\right)=N\left(x;m\left(x_{k}\right),Q\right),$$

where $m\left(x_{k}\right)={\left[{\left(F\left(w_{k}\right){\tilde{x}}_{k}\right)}^{\prime },w_{k}\right]}^{\prime }$, $Q=\mathrm{diag}\left(\left[\sigma_{w}^{2}GG^{\prime },\sigma_{u}^{2}\right]\right)$, and the parameters

Fw=1sinwTsinwTcoswTcoswT0−1−coswT1−coswTww0coswT0−sinwT01−coswT1−coswTww1sinwTsinwTww0sinwT0coswT,G=T2T2220T00T2T2220T,

where T=1s is the sampling time. The standard deviation of the process noise $\sigma_{w}=5\;m/s^{2}$, the standard deviation of the turn rate noise $\sigma_{u}=\frac{\pi }{180}\;\mathrm{rad}/s$. The survival probability (for prediction) is set to be constant $p_{s}=0.99$. For the birth process, we follow the model in [20].

**Figure 5:**
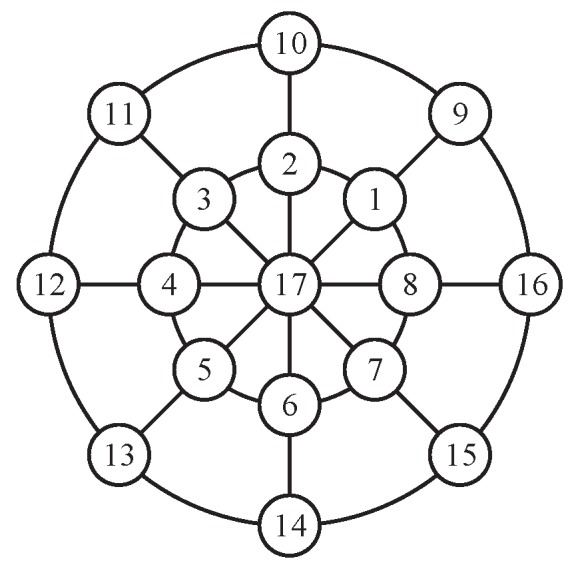
The admissible positions of the sensor.

The initial position of the mobile sensor is (−2000m,−2000m). We assume that the sensor control process is an ideal control process in Section 2.2. In addition, the admissible control actions is calculated by Equation (11). The number of sensor s=1. Given the previous position of the sensor $s_{k-1}=\left(x_{s_{k-1}},y_{s_{k-1}}\right)$, the admissible positions of sensor follows

$$S_{k}={\left\{\left(x_{s_{k-1}}+j\frac{v}{N_{R}}\cos\left(l\frac{2\pi }{N_{\theta }}\right),y_{s_{k-1}}+j\frac{v}{N_{R}}\sin\left(l\frac{2\pi }{N_{\theta }}\right)\right)\right\}}_{l=1}^{N_{\theta }},$$

for $j=\left\{0,1,2\right\}$ and $N_{R}=2$. v=50m/s represents for the maximum speed of the sensor and $N_{\theta }=8$ is the directions. Figure 5 shows seventeen admissible positions of the mobile sensor. We use $s_{k}=\left(x_{s_{k}},y_{s_{k}}\right)$ to denote the current position of the sensor.

The location-dependent probability of detection is

$$p_{D,k}\left(x_{k}\right)\propto p_{D\max}\cdot N\left(x_{k};s_{k},\mathrm{diag}{\left(\left[4000,4000\right]\right)}^{2}\right),$$

where $p_{D\max}=0.98$ is its peak value. If detected, each target shall produce one observation $z={\left[\theta ,r\right]}^{\prime }$, namely bearing and range measurement.

The likelihood is calculated as

$$g_{k}\left(z|x_{k},s_{k}\right)=N\left(z;\mu \left(x_{k},s_{k}\right),R\right),$$

where $\mu \left(x_{k},s_{k}\right)=\left[\arctan\left(\frac{x_{k}-x_{s_{k}}}{y_{k}-y_{s_{k}}}\right),\sqrt{{\left(x_{k}-x_{s_{k}}\right)}^{2}+{\left(y_{k}-y_{s_{k}}\right)}^{2}}\right]$ and $R=\mathrm{diag}\left({\left[\sigma_{\theta }^{2},\sigma_{r}^{2}\right]}^{\prime }\right)$. We set $\sigma_{\theta }=\frac{\pi }{180}\mathrm{rad}$ and $\sigma_{r}=5\;m$.

Recalling the clutter model in the update step, it follows a Poisson RFS with intensity $\kappa_{k}\left(z\right)=\lambda_{c}U\left(Z\right)$, where U(Z) denotes a uniform density on the disc of radius 2000 m, and we set $\lambda_{c}=8\times 10^{-4}{\left(\mathrm{radm}\right)}^{-1}$ for an average of 10 clutters per scan.

The particle implementation of σ-GLMB filter is employed in the simulations, for its flexibility in dealing with the nonlinear tracking problems. The setup of particle filter is 1000 particles per target, and 500 particles is the threshold on effective number of particles before resampling.

### 6.2. Results and Analysis

**Figure 6:**
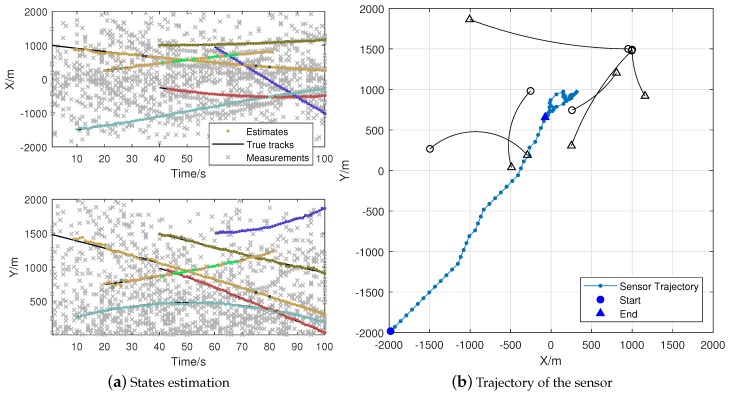
Dual sensor control algorithm with PAPD and PDST.

**Figure 7:**
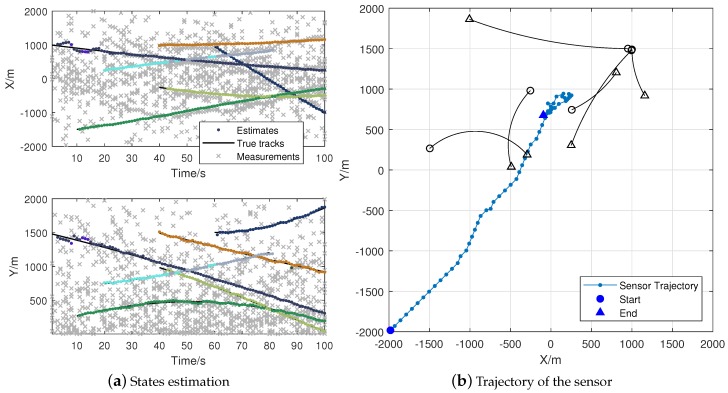
Dual sensor control algorithm with PDST and PDST.

In this part, we use the “double controller” to denote the algorithm with a dual sensor control scheme. Figure 6 is a single run of the Algorithm 1 with metric pair PAPD-PDST. We assign each track of the estimation results with different colors in Figure 6a. Figure 6b shows the trajectory of the sensor, and each point denotes the sensor position after ${\mathrm{Controller}}_{2}$. Figure 7 provides the results of Algorithm 1 with metric pair PDST-PDST. It shows that the proposed algorithms can obtain acceptable estimation results while achieving the task of tracking. Both of the algorithms can drive the sensor to the center of targets.

**Figure 8:**
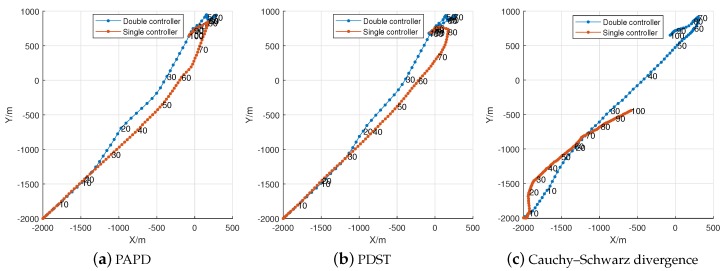
The expected trajectory of sensor (100 Monte Carlo trials).

To analyze the statistically characteristics of the algorithms, we performed 100 Monte Carlo simulations on each of them. The expected sensor trajectories with timestamps after employing the sensor control processes are given in Figure 8a,b. For comparison, we use the sensor control method based on Cauchy–Schwarz divergence, and the results are provided in Figure 8c. The trajectories of the first two pictures are quite similar, while exhibiting significant difference with Figure 8c. Approximate analysis based on the timestamps, we find that the proposed algorithms with double controller are more effective. The tracking results of the Cauchy–Schwarz divergence-based algorithms are tardy, especially for its single controller situation. Fortunately, the introduction of double controller has improved the efficiency of tracking.

**Figure 9:**
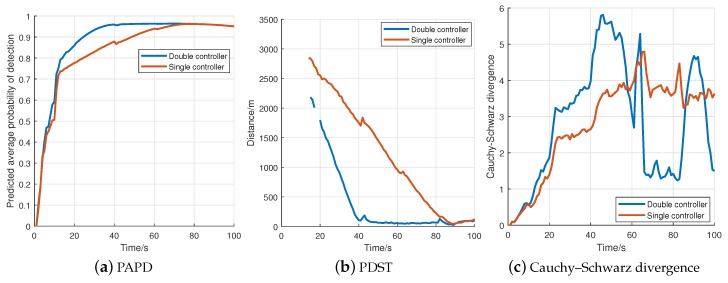
The average evaluation values for ${\mathrm{Controller}}_{1}$ (100 Monte Carlo trials).

The average values of each metric during the ${\mathrm{Controller}}_{1}$ are drawn in Figure 9. Figure 9a shows the tendency of average probability of detection gradually increases until it reaches its peak (approximately 40 s for double controller and 70 s for single controller). Figure 9b shows the tendency of average distance between sensor and targets gradually decreases until the sensor reaches targets’ center (approximately 45 s for double controller and 90 s for single controller). No obvious trend is indicated in Figure 9c. Note that the missing part in Figure 9b (first 20 s) is because no target is extracted in some Monte Carlo trials.

**Figure 10:**
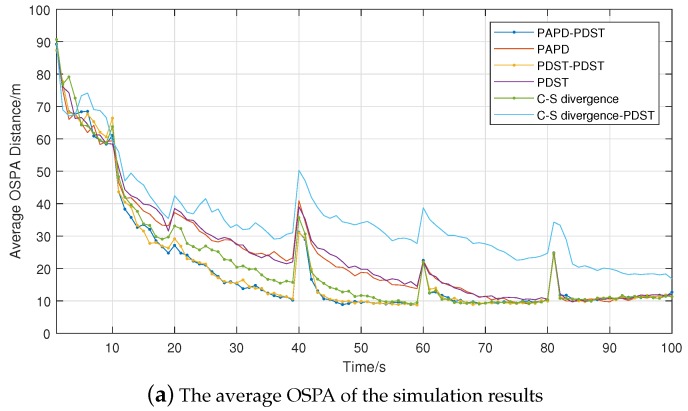

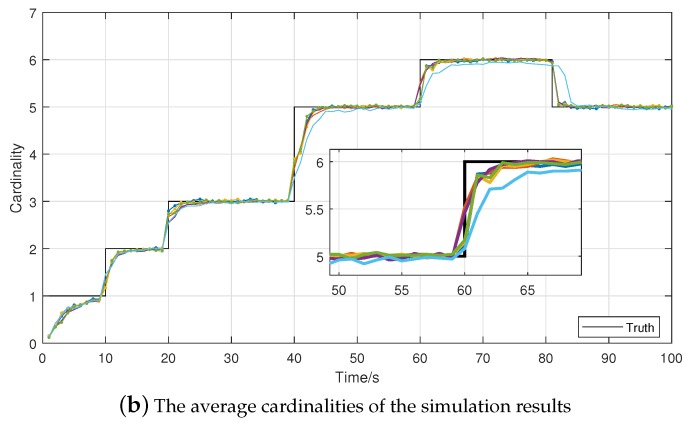
Performance evaluations (100 Monte Carlo trials).

To evaluate the performance of different algorithms in multi-target state estimation, the optimal sub-pattern assignment (OSPA) [22] metric is utilized, the calculation is carried out by Equation (29). Figure 10a shows the results of OSPA (with parameters, c = 100 and p = 1), and it indicates that the errors of double controllers are smaller. Figure 10b shows the cardinality of estimations compared to the truth. It indicates a similar performance, except for the single controller with a Cauchy–Schwarz divergence (C–S divergence). Whenever the number of targets changes, the OSPA errors will suddenly increase (at 20, 40, 60, 80 time steps) and eventually settle to a certain value.

Table 1 shows the average computing time for 100-time steps. It indicates that a double controller with the PAPD metric uses the least computing time, approximately 8.34 s per step. For one step, the PDST-based algorithms need about 44 s and Cauchy–Schwarz divergence-based algorithms needs 50 s. This result validates the effectiveness of the proposed PAPD metric. Actually, Table 1 indicates that double sensor control algorithms require less computing time overall than that of a single controller. This is because the additional controller can increase the overall tracking efficiency by driving the sensor to a location with a higher detection probability. The simulations are implemented in MATLAB R2017b (The MathWorks, Inc., Natick, MA, USA) on a desktop computer with an Intel Core i5-4570 CPU (Santa Clara, CA, USA) and 4 GB of RAM.

### 6.3. Further Discussion

To investigate the adaptability of the dual sensor control algorithm with PAPD and PDST, another commonly used location-dependent detection function is introduced,

$$p_{D}\left(x,s_{k}\right)=\left\{\begin{array}{ll} p_{D\max} & \mathrm{if}\;∥x-s_{k}∥\leq R\\ \max\left\{0,p_{D\max}-h\cdot \left(∥x-s_{k}∥-R\right)\right\} & \mathrm{otherwise}, \end{array}\right.$$

where $p_{D\max}=0.98$, R=300m, and $h=1.25\times 10^{-4}\;m^{-1}$.

**Figure 11:**
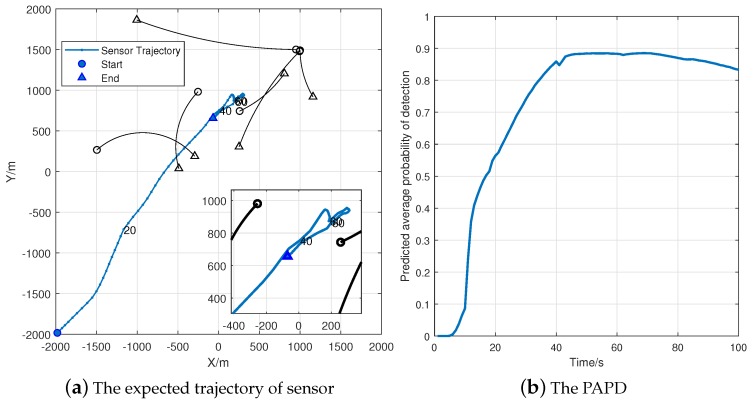
Simulation results using the alternative $P_{D}$ function (100 Monte Carlo trials).

Figure 11a indicates that the expected trajectory is similar to the double controller in Figure 8a. In addition, Figure 11b exhibits a significant tendency of increasing in PAPD during the first 40 time steps. Based on these simulation results, the adaptability of Algorithm 1 is verified.

## 7. Conclusions

A dual sensor control scheme for multi-target tracking was proposed in the context of POMDPs with FISST. The proposed scheme does not rely on a specific filter, and the existing evaluation function can be applied to the dual sensor control scheme straightforwardly. Typically, a dual sensor control algorithm is characterized by the metric pair. From a task-driven perspective, two novel metrics were developed. The PDST metric is based on an understanding of multi-target tracking. In addition, the motivation of the PAPD metric is to improve the efficiency. Simulation results demonstrated that the proposed dual sensor control scheme can improve the multi-target state estimation accuracy and the overall efficiency. Moreover, the algorithm that uses the recommended metric pair (PAPD-PDST) has shown excellent performance and adaptability. For the further study, we will apply these methods to the multi-sensor and more complex scenarios.

## Figures and Tables

**Table 1:** The average computing time of the algorithms.

	PAPD	PDST	C–S Divergence
Double controller	$8.34\times 10^{2}$ s	$4.47\times 10^{3}$ s	$5.00\times 10^{3}$ s
Single controller	$8.85\times 10^{2}$ s	$4.73\times 10^{3}$ s	$5.80\times 10^{3}$ s

## Abbreviations

The following abbreviations are used in this manuscript:

POMDPs	partially observed Markov decision processes
FISST	Finite Set Statistics
MTT	Multi-Target Tracking
ICP	Ideal Control Process
GLMB	Generalized Labeled Multi-Bernoulli
RFS	Random Finite Set
PDST	Posterior Distance between Sensor and Targets
PAPD	Predicted Average Probability of Detection
OSPA	Optimal Subpattern Assignment
